# MILLET BASED NUTRITIONAL SUPPLEMENT FOR DIARRHEAL EPISODE IN PROTEIN ENERGY MALNOURISHED CONDITION: AN EXPERIMENTAL APPROACH IN CASTOR OIL MODEL

**DOI:** 10.1590/S0004-2803.24612024-098

**Published:** 2025-04-04

**Authors:** Sachin V TEMBHURNE, Mansi S JAGDALE, Payal KATE, Ziyaurrahman AR

**Affiliations:** 1 Department of Pharmacology, AISSMS College of Pharmacy, Kennedy Road, Pune 411001, India.; 2 Pharmacology Department, Allana College of Pharmacy, Pune, India.

**Keywords:** Castor oil, diarrhoea, nutritional supplement, fermentation, bovine colostrum, Óleo de rícino, diarreia, suplemento nutricional, fermentação, colostro bovino

## Abstract

**Background:**

Diarrhea is a gastrointestinal transit disorder and mostly seen in malnourished children’s as per WHO report. Malnourished individuals are found to be associated with compromised immunity and lack of nutrients, which makes person susceptible to diarrhoea.

**Objective:**

For maintaining the gut health adequate and balance nutrition is essential. In this study, both fermented and non-fermented nutritional supplement was formulated and evaluated against castor-oil induced diarrhoea.

**Methods:**

Two groups of rats initially fed a 2% protein-deficient diet for ten weeks. After this period, one group received a diet enriched with nutritional components blended with fermented bovine colostrum, while the other group received a diet with non-fermented nutritional components. At the end of 20th week, castor oil was given to all animals except the control group to induce diarrhoea. Subsequently, these rats were subjected to various assessments including time of onset of the first diarrheal stool (min), faecal weight, faecal score, number of wet stools, intestinal fluid accumulation and Histopathological examination. DPPH radical-scavenging activity of nutritional blend was also determined.

**Results:**

The undernourished rats fed with non-fermented and fermented diet showed delayed the onset of diarrhea and reduction of weight stool, the decrease in the frequency and severity of defecation as well as significantly protected against the intestinal fluid accumulation as compare to negative control groups. The results showed that both the fermented and non-fermented blended composition exhibited antioxidant activity. The intestine of undernourished rats fed with fermented nutritional diet showed the absence of infiltration and improved villi structure.

**Conclusion:**

The study presents promising evidence of the potential benefits of the formulated nutritional compositions in alleviating the castor oil-induced diarrhea in undernourished wistar rats. The antioxidant activity, anti-diarrheal effects and improvements in gut histology suggest that, the nutritional compositions could be explored further as natural interventions for gastrointestinal health.

## HIGHLIGHTS

• Diarrhea is common in malnourished children due to compromised immunity and lack of nutrients.

• Rats were fed a protein-deficient diet (2%) for 10 weeks. One group received fermented bovine colostrum, while the other received non-fermented nutritional components.

• Diarrhea was induced in the animals, and they were evaluated for the onset of diarrhea, fecal weight, frequency, intestinal fluid accumulation, and histology.

• Both supplements delayed the onset of diarrhea, reduced its severity, and exhibited antioxidant activity.

• The nutritional compositions have potential benefits as natural interventions for intestinal health.

## RESUMO

**Contexto:**

A diarreia é um distúrbio do trânsito gastrointestinal e, segundo relatório da OMS, é frequentemente observada em crianças desnutridas. Indivíduos desnutridos apresentam imunidade comprometida e falta de nutrientes, tornando-os mais suscetíveis à diarreia.

**Objetivo:**

Para manter a saúde intestinal, uma nutrição adequada e equilibrada é essencial. Neste estudo, foi formulado e avaliado um suplemento nutricional, fermentado e não fermentado, contra a diarreia induzida por óleo de rícino.

**Métodos:**

Dois grupos de ratos foram inicialmente alimentados com uma dieta deficiente em proteínas (2%) por dez semanas. Após esse período, um grupo recebeu uma dieta enriquecida com componentes nutricionais misturados com colostro bovino fermentado, enquanto o outro grupo recebeu uma dieta com componentes nutricionais não fermentados. Ao final da 20ª semana, foi administrado óleo de rícino a todos os animais, exceto ao grupo controle, para induzir diarreia. Subsequentemente, esses ratos foram submetidos a várias avaliações, incluindo o tempo de início do primeiro episódio de fezes diarreicas (em minutos), peso fecal, escore fecal, número de fezes úmidas, acúmulo de fluidos intestinais e exame histopatológico. A atividade sequestradora de radicais DPPH da mistura nutricional também foi determinada.

**Resultados:**

Os ratos desnutridos alimentados com dietas fermentadas e não fermentadas mostraram atraso no início da diarreia, redução do peso das fezes, diminuição na frequência e gravidade da defecação, bem como proteção significativa contra o acúmulo de fluidos intestinais em comparação aos grupos controle negativo. Os resultados mostraram que ambas as composições misturadas, fermentadas e não fermentadas, exibiram atividade antioxidante. O intestino dos ratos desnutridos alimentados com a dieta nutricional fermentada mostrou ausência de infiltração e melhora na estrutura das vilosidades.

**Conclusão:**

O estudo apresenta evidências promissoras dos benefícios potenciais das composições nutricionais formuladas no alívio da diarreia induzida por óleo de rícino em ratos Wistar desnutridos. A atividade antioxidante, os efeitos antidiarreicos e as melhorias na histologia intestinal sugerem que essas composições nutricionais podem ser exploradas como intervenções naturais para a saúde gastrointestinal.

## INTRODUCTION

Diarrhoea is a gastrointestinal transit disorder with features such as frequent evacuation more than three times per day, stools of abnormal form, soft, even fluid, and unusually large volume. WHO stated that malnourished children are more prone to incidence of diarrhea^1^. Malnourished individuals, marked by compromised immune systems and diminished physiological reserves, are particularly susceptible to the deleterious effects of diarrheal episodes. Diarrhoea found to be associated with inadequate absorption of nutrient, maldigestion and loss of nutrients. They have bidirectional relationship. Adequate and balanced nutrition is important for maintaining the gut health. Improper nutrition results in a loss of intestinal function decreased flow to intestine and altered villous architecture. Malnutrition found to have increased risk of infections in individuals^2^. Additionally, malnutrition has been found to link with decreased level of antioxidant nutrients like vitamin A and vitamin E. Also low levels of glutathione peroxidase found. Malnourished children also showed increased level of TBARS in plasma^3^ ^,^ ^4^. Because GIT infections are potent oxidizing agent this states that malnutrition individuals with depleted antioxidant system prone to diarrhoea associated complications^5^.

Fermented foods have been adopted as traditional foods all over the world, with many cultures and societies using fermented foods to alleviate diarrhoea, especially among children^6^ ^,^ ^7^. This ancient food processing approach is still used as a food preservation method both domestically and commercially, increasing the shelf life of foods and beverages^8^. Additionally, fermentation found to increase the antioxidant properties of the food which can help to neutralize the oxidising species^9^. Improper nutrients can affect the integrity of the gastrointestinal tract, leading to a compromised gut barrier function. This can result in the entry of pathogens, toxins, and other harmful substances into the bloodstream, triggering an immune response. Inflammation and immune activation contribute to the production of ROS and reactive nitrogen species (RNS) as part of the body’s defence mechanisms. However, in malnourished individuals with inadequate antioxidant defences, the excessive ROS and RNS production can lead to oxidative stress and damage to gut tissues^10^ ^,^ ^11^. Proper diet can modulate the gut and aid in maintain the gut health^12^

**FIGURE 1:**
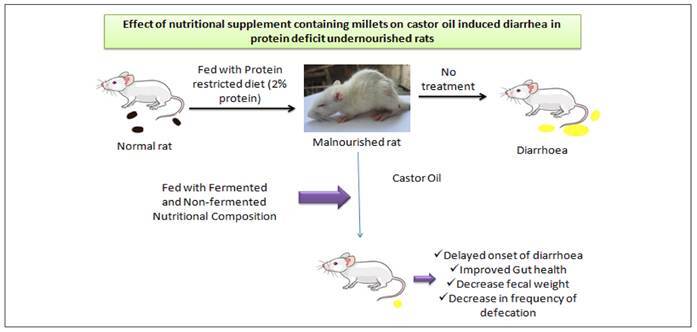
Study design of nutritional supplement in castor oil induced diarrhoea in malnutrition.

Thus, in the present study, we have studied the effect of nutritional supplement on diarrhoea in protein deficit undernourished condition in rats and to study how the different dietary interventions could be protective against castor oil induced diarrhoea. The overall design of the present investigation given in Figure 1.

## METHODS

### Selection and procurement of plant materials

Pearl millet (*Pennisetum glaucum*), Finger millet (*Eleusine coracana*), Red lentils (*Lens culinaris*), Moong beans (*Vigna radiata*), Flax seed *(Linum usitatissimum*) and Red rajma (*Phaseolus vulgaris*) were procured from Satvyk Organic Food Store, Pune. Shatavari (*Asparagus racemosus*) were procured from Manikarnika Aushadhalay, Chinchwad, Maharashtra India.

### Collection of bovine colostrum

BC (collected during the first 24 h postpartum) was obtained from a local dairy farmer. Immediately after collection, BC was stored (−20°C) for 72 h. After that following lyophilization, BC was kept at room temperature in plastic zipper bags and in sealed polystyrene boxes until it was used for oral supplementation. 

### Preparation of fermented and non-fermented blended nutritional composition

The millet, red lentils, moong beans, and red rajma underwent a visual inspection to ensure there were no visible impurities. Subsequently, they were soaked in water for 12 hours. Following the soaking period, the damp seeds were placed in a cotton cloth and placed in a dark room to facilitate proper germination. Upon completion of the germination process, all the seeds were dried using sunlight and air. Once dried, they were milled into fine powders. The quantities of each seed powder were measured as specified in the table and mixed in a Mason jar. To this mixture, approximately 500 mL of distilled water is added. Thorough mixing is performed to ensure uniform distribution of the ingredients. Subsequently, the lid is tightly sealed, and the jar was allowed to ferment for a period of approximately 72 hours.

During the fermentation process, the pH was measured at intervals of 24 hours. The process was continuing until the pH reaches to value of pH 4.0. Once fermentation is completed and the desired pH is achieved, the mixture is formed into pellets using a 1:1 ratio, specifically combining 50% of the nutritional blend with 50% of the protein-restricted diet.

For non-fermented composition, similar procedure was followed as fermented composition except the fermentation step. The pellets of final nutrition composition were made in the 1:1 ratio, specifically combined with 50% of the nutritional blend (Table 1) with 50% of the protein-restricted diet (Table 2). 

**TABLE 1:** Composition of Fermented blend.

Ingredients	Quantity (per kg)
Lens culinaris	180 g
Vigna radiata	100 g
Phaseolus vulgaris	180 g
Pennisetum glaucum	200 g
Eleusine coracana	70 g
Linum usitatissimum	100 g
Asparagus racemosus	70 g
Bovine colostrum	100 g

**TABLE 2:** Composition of Protein restricted diet.

Ingredient	Standard diet AIN 93 G diet (g/kg)	Low Protein and folate diet (2%) (g/kg)
Casein	200	23
Corn starch	397.486	489.7
Dextrinized Cornstarch	132	132
Sucrose	100	200
Cellulose	50	65
Soyabean oil	70	70
Vitamin mix	10	0
Mineral mix	30	0
Methionine	3	3
Choline	2.5	2.5

### Preparation of modified AIN-93G protein restricted diet (2%)

The development of nutritional composition of the low protein diet (LPD) according to the specifications outlined in National Research Council (US) Subcommittee on Laboratory Animal Nutrition^13^. The protein content was deliberately limited to 2% based on the documented results of comparable studies^14^. The ingredients were combined and pellets were made and dried. (TABLE 2)

### Experimental design

Thirty-two female Wistar rats with body weights ranging of 200-230 g were sourced from the Animal House of AISSMS College of Pharmacy, Pune. The rats underwent a one-week acclimatization period in controlled laboratory conditions, maintained at a temperature of 25±2°C. The rats were then randomly assigned to one of four groups. Control group animals fed with a standard pellet diet with 20% protein throughout the entire study; the negative control group fed with a low protein diet with 2% protein throughout the study; the experimental group 1 animals fed with protein restricted diet for 10 weeks and then fed with non-fermented nutritional diet for next 10 weeks; the experimental group 2 fed with protein restricted diet for 10 weeks and then fermented nutritional diet for 10 weeks. 

At the end of 20th week, castor oil (5 mL/kg b.w., p.o.) was given to all animals except the control group and evaluate the following parameters. 

Post diarrhoea induction: animals of all groups were examined for the presence of diarrhoea confirmed by defecation of watery stool from the anus. All the animals were observed for a period of 6 hours for the presence of characteristic diarrhoea droopings which is recorded with the help of predetermined scoring index (Di Carlo et al., 1993)^15^ as follows: (++) for copious, (+) for mild and 0 for lack of diarrhoea. The presence of stool or any fluid materials that stained the blotting paper placed under each cage lined with the floor was considered diarrhoea. Diarrhoea was defined as faeces that were unformed, muddy, or watery. The time before the first defecation is known as ‘latent period’. Total faecal output and diarrheic faeces (muddy or watery faeces) excreted by the experimental animal for 4 hours after the latent period were determined.

The time period from week 10 to week 20 has been utilised for increasing probiotic bacteria in rat guts. This duration time was not taken into consideration for data collection.

### Enterpooling determination

The intestinal fluid accumulation was determined according to Carlo et al. After 2 hrs of administration of castor oil, animals were sacrificed using chloroform. After scarification, the small intestine was removed at pyloric end after ligation and ileocaecal junction. The contents were expelled out in cylinder and weighed. The small intestine was remeasured, and the differences in organ weights between full and empty organs were calculated.

### DPPH radical-scavenging activity

Sample stock solutions (1.0 mg/mL) of nutritional composition were diluted to final concentrations of 80, 160, 240, 320, 400 µg/mL in methanol. In brief 1.2 mL of 0.1 Mm DPPH in methanol solution was mixed with 0.8 ml of sample of different concentrations and allowed to react at room temperature. After 30 min, the absorbance values were measured at 518 nm and converted into the percent inhibition using below formula. The standard used was ascorbic acid. DPPH solution (1.2 mL; 0.3 mM) plus methanol (0.8 mL) was used as a negative control. 

DPPH Scavenging effect % inhibition= 

Where, 

A0=the absorbance of control, 

A1=the absorbance of test sample.

### Statistical analysis

Data were analyzed by One-way analysis of variance (ANOVA) followed by Tukey-Kramer test at *P*<0.05, *P*<0.01, *P*<0.001. All the values were expressed as Mean ± SD. 

### Ethical Statement

This research was approved by the Institutional Animal Ethics Committee of AISSMS College of Pharmacy registered with CPCSEA New Delhi register no.257/P0/ReBi/S/2000/CPCSEA.

## RESULTS

### DPPH radical scavenging activity of nutritional composition

The IC 50 of standard ascorbic acid was found to be 34.57 μg/mLwhereas for fermented food it was found to be 266.45 μg/mL. The IC 50 of non-fermented food was found to be 612.5 μg/mL. The % radical scavenging activity shown in Figure 2.

**FIGURE:**
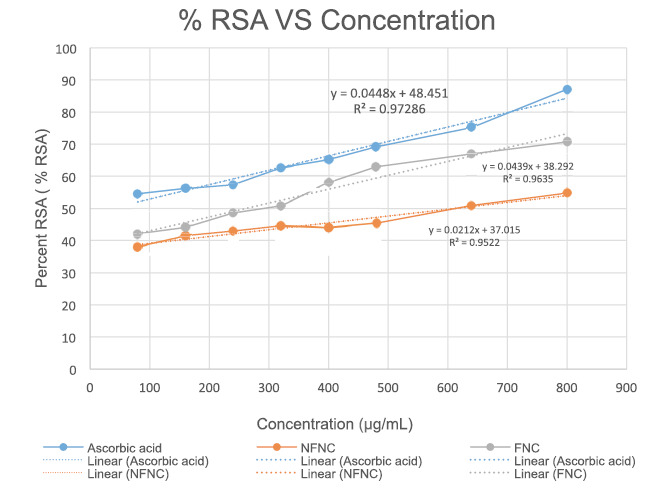
2. Percent DPPH radical scavenging by fermented and non-fermented nutritional composition.

### Effect of nutritional composition on castor oil-induced diarrhoea in rats

The oral administration of castor oil (5 mL/kg, b.w., p.o.) in protein deficit undernourished rats showed production of copious diarrhea (number of wet stools 7.2±0.83). The undernourished rats fed with non-fermented and fermented diet showed delayed the onset of diarrhea (#*P*<0.05, ##*P*<0.01) and reduction of weight stool as well as the decrease in the frequency and severity of defecation (22.22 % and 41.66 % respectively). The results shown in Table 3.

**TABLE 3:** Effect of formulated nutritional composition on castor oil induced diarrhoea (n=5).

Groups	Time of onset of the first diarrheal stool (min)	Faecal score	Faecal weight	Number of wet stools	Percentage protected (No. of wet stools)
Control	0	1.0±0	1.13±0.30	0±0	0
PRD + Castor oil	56.6±6.18^***^	2.4±0.54^***^	2.42±0.22^***^	7.2±0.83***	0
NFNC+ Castor oil	79.4±9.5^#^	2.2±0.44^#^	1.9±0.22^##^	5.6±0.54^#^	22.22%
FNC+ Castor oil	89.8±12.31^##^	1.6±0.54^##^	1.86±0.11^##^	4.2±0.83^#^	41.66%

PRD: protein restricted diet, NFNC: non-fermented nutritional composition, FNC: fermented nutritional composition. All values are expressed as Mean ± SD, (n=5); *P<0.05; **P<0.01; ***P<0.001 as compared to control; #*P*<0.05; ##*P*<0.01, ###*P*<0.001 as compared to negative control on 2% protein restricted diet. Statistically analysed by one-way ANOVA followed by Tukey-Kramer Multiple Comparisons Test.

### Effect of nutritional composition on castor oil induced enterpooling

As shown in the Table 4, the undernourished rats fed with non-fermented and fermented nutritional diet significantly protected (36.22% and 43.30% respectively) against the intestinal fluid accumulation induced by castor oil intoxication. The secretions obtained in animals fed with nutritional composition are more viscous than the protein restricted diet group. Thus, nutritional composition protects against castor oil-induced enterpooling.

**TABLE 4:** Effect of nutritional composition on castor oil induced enterpooling (n=5).

Groups	Intestinal fluid (mL)	Inhibition of Intestinal fluid volume (mL) (%)
Control	0.55±0.07	-
PRD + Castor oil	2.54±0.43***	-
NFNC+ Castor oil	1.62±0.37#	36.22%
FNC+ Castor oil	1.44±0.35#	43.30%

All values are expressed as Mean ± SD, (n=5); *P<0.05; **P<0.01; ***P<0.001 as compared to control; #*P*<0.05; ##*P*<0.01; ###*P*<0.001 as compared to negative control on 2% protein restricted diet. Statistically analysed by one-way ANOVA followed by Tukey-Kramer Multiple Comparisons Test.

### Histopathology of intestine

The intestine of normal control rats exhibits characteristic morphology of villi. The intestine of protein deficit undernourished rats showed marked goblet cell hyperplasia in the villi, degenerative and disruptive changes in the mucosal epithelium in the villus, Necrotic changes in the epithelium of villi with short and reduced length of villi with infiltration of mononuclear inflammatory cells in the mucosa and submucosa were seen. 

The results of intestine of undernourished rats fed with non-fermented nutritional diet showed hyperplasia of goblet cells, the villi structure was improved than the rats fed with protein deficit diet (undernourished animal) but still some degree of disruptive changes was seen. 

**FIGURE 3:**
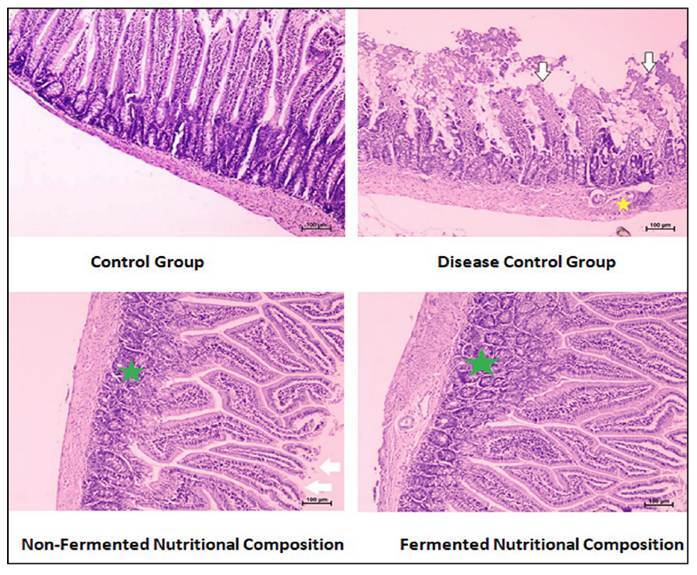
Histopathological examination of intestine. White arrow indicates degenerative and disruptive changes in the mucosal epithelium in the villus. Necrotic changes in the epithelium of villi with short and reduced length of villi, green star indicates goblet cell hyperplasia in the villi, yellow star indicates with infiltration of mononuclear inflammatory cells in the mucosa and submucosa.

The intestine of undernourished rats fed with fermented nutritional diet showed the absence of infiltration and improved villi structure as well as length but still goblet hyperplasia was observed. Figure 3 shows the histopathological findings of intestine. 

## DISCUSSION

The present study investigated the effects of a nutritional supplement, including the fermented and non-fermented blended composition, on castor oil-induced diarrhea in protein deficit undernourished rats. The findings of the study provide the valuable insights into the potential benefits of these compositions on gastrointestinal health.

The study assessed the DPPH radical-scavenging activity of the nutritional compositions. The DPPH assay is commonly used to evaluate the antioxidant potential of compounds^16^. The results showed that both the fermented and non-fermented blended composition exhibited antioxidant activity, as evidenced by their ability to scavenge DPPH radicals. The IC50 values, which represent the concentration at which 50% of the radicals are scavenged, were higher for the fermented blend compared to the non-fermented blend (266.45 μg/mL, 612.5 μg/Ml). The results of DPPH scavenging activity is supported with the similar reported finding, in which the fermented cereals were shown to have significant increase in antioxidant activity than non-fermented^17^. These finding suggest that, the fermentation process may have enhanced the antioxidant activity of the nutritional composition. The nutritional composition rich with antioxidant components would be helpful in management of oxidative stress which is implicated in various gastrointestinal disorders.

Considering the potential antioxidant effects in nutritional composition, our study further undertaken to evaluate its potency in castor oil induced diarrhoea in protein deficient undernourished condition in rats. The finding shows significant diarrhoeal symptoms in protein deficit undernourished rats, the symptoms included the increased faecal score, faecal weight, and wet stools. The active metabolite of castor oil, ricinoleic acid, stimulates peristaltic movement in the small intestines, resulting in alterations in intestinal mucosa permeability to electrolytes^18^. In malnourished individuals these conditions are already exaggerated due to improper nutrition and decreased protein content which results in gut barrier dysfunction. While the finding of the non-fermented and fermented nutritional diet, demonstrated a reduction in these symptoms compared to the undernourished rat fed with protein deficit diet alone. These finding suggests that, the both nutritional compositions have potent anti-diarrheal effects. However, the fermented blend exhibited a more pronounced reduction in symptoms compared to the non-fermented blend. This difference could be attributed to the presence of bovine colostrum in fermented composition. The results of the study are supported with reported finding on bovine colostrum which showed improve in the gut barrier function and found to reduce the intestinal damage as well as stool frequency^19^ ^,^ ^20^ results also correlate with another reported study in which the diet containing yoghurt, lentils found to have alleviate the conditions of diarrhoea^21^.

Histopathological analysis of the intestinal tissue provided additional insights into the effects of the nutritional compositions. The intestine of undernourished rat fed with protein deficit diet displayed disrupted villi structure, goblet cell hyperplasia, and inflammatory cell infiltration. These changes are indicative of intestinal damage and inflammation associated with diarrhea. It is obvious that excessive production of free radicals negatively impacts the cells and may play a significant role in the deterioration of proteins, lipids, and cell structures^22^ and also plays important role in diarrhoea^23^ and gastric ulcer^24^. The finding of undernourished rats fed with nutritional composition diet especially the fermented nutritional composition, showed improvements in villi structure, reduction in goblet cell hyperplasia, and absence of inflammatory cell infiltration. These findings suggest that the nutritional compositions, particularly the fermented blend, have the potential to mitigate the histological damage associated with diarrhea. 

The study presents promising evidence of the potential benefits of the formulated nutritional compositions in alleviating the castor oil-induced diarrhea in undernourished wistar rats. The antioxidant activity, anti-diarrheal effects, and improvements in gut histology suggest that, the nutritional compositions could be explored further as natural interventions for gastrointestinal health. Furthermore, the fermented diets can be used to alleviate the diarrhoeal incidences in malnourished condition. While the further detail study needed to understand the mechanisms behind the potential effects of novel nutritional composition and to assess their relevance in human health and disease.
